# Ethereal Instillations into the Ear for the Cure of Deafness

**Published:** 1860-12

**Authors:** 


*Ethereal Instillations into the Ear for the Cure of Deafness.*—The excellent report read by M. Meniere, Physician to the Deaf and Dumb Asylum in Paris, at a late meeting of the Academy, has completely set at rest the question of the efficacy or inefficiency of ethereal instillations into the ear for the cure of surdo-mutity. M. Meniere availed himself of the advantages afforded by his position at the Asylum, and instituted a series of experiments with a view to the solution of what, strange to say, was, and is still in many minds, an undecided question, and came to the conclusion that sulphuric ether exerts *no action whatever* upon the auditory senses of those congenitally deaf and dumb. M. Meniere might have been entitled to carry his condemnation still further, but said that, in his character of scientific experimentalist he was bound scrupulously to respect the limits he had himself imposed upon his researches.—*Bos. Med. and Sur. Jour.*

